# Exploring the Antibacterial and Biosensing Applications of Peroxidase-Mimetic Ni_0.1_Cu_0.9_S Nanoflower

**DOI:** 10.3390/bios12100874

**Published:** 2022-10-15

**Authors:** Li Liu, Yayu Lai, Jinming Cao, Yu Peng, Tian Tian, Wensheng Fu

**Affiliations:** 1 Chongqing Key Laboratory of Green Synthesis and Applications, College of Chemistry, Chongqing Normal University, Chongqing 401331, China; 2 The Department of General Practice, The 958th Hospital of Chinese People’s Liberation Army, Chongqing 400000, China

**Keywords:** peroxidase mimicase, reactive oxygen species, antibacterial application, biosensing, copper-containing nanozymes

## Abstract

Nanozymes, as artificial enzymes with the biological action of natural enzymes, have enormous potential in the fields of disease diagnosis, bacteriostasis, biosensing, etc. In this work, the Ni_0.1_Cu_0.9_S nanoflower was successfully synthesized through a one-step hydrothermal method. A combined strategy of Ni doping and morphology design was employed to adjust its electronic structure and active sites, endowing the Ni_0.1_Cu_0.9_S nanoflower with excellent peroxidase-like activity. Therefore, it can catalyze the decomposition of H_2_O_2_ to generate •OH with higher antibacterial activity, establishing a broad-spectrum antibacterial system based on the Ni_0.1_Cu_0.9_S nanoflower against *E. coli* and *S. aureus*, which avoids the harm of a high concentration of H_2_O_2_. Additionally, the colorless substrate TMB can be catalytically oxidized into blue ox-TMB via •OH. As a result, a colorimetric technique with rapid and accurate detection of ascorbic acid (AA) by the unaided eye was designed, in view of the specific inhibition effect towards the oxidation of TMB. This detection platform has a wide linear range (10~800 μM) with a low limit of detection (0.84 μM) and exhibits a satisfactory selectivity toward the detection of AA. This study sheds new light on the application of copper-containing nanozymes in the fields of biomedicine and bioassay.

## 1. Introduction

A natural enzyme is a kind of biocatalyst that can catalytically mediate various physiological processes in living organisms [1,2]. The reactions involving natural enzymes are highly efficient and specific [3]. Regrettably, owing to their easy deactivation, difficulties in purification, storage and recovery, high price and harsh operating conditions, natural enzymes are not suitable for reactions in a nonphysiological environment, which severely limits their large-scale application [4]. As opposed to the drawbacks of natural enzymes, artificial enzymes (also known as nanoenzymes) with the merits of robust stability, simple preparation, high reliability, low cost and tunable catalytic properties can be used in harsh reaction situations [5,6]. Consequently, nanoenzymes, especially those that could promote the production of reactive oxygen species (ROS), can be wildly applied to disease treatment [7,8], biological detection [9,10], bacteriostasis [1,11,12,13] and other fields [14].

In recent years, the outbreak of infectious disease brought by bacteria has become a public health risk worldwide, which seriously threatens human life and health [15,16]. Bacterial infections are usually treated with antibiotics, which could selectively inhibit and kill bacteria by restraining DNA replication/repair and protein synthesis [17,18]. However, the overuse of antibiotics has led to inflammatory diseases related to antibiotic-resistant strains [19]. This situation reduces the effectiveness of antibiotic treatment and increases the number of infection-related deaths each year. Therefore, the development of a functionalized antibacterial material with an enzyme-like performance that can not only play the role of an antibiotic but also avoid the resistance of bacteria has become a hot topic. Previous studies have shown that the bactericidal mechanism of many antibiotics is to induce oxidative stress by ROS (e.g., •OH, •O^2−^, H_2_O_2_) production through the Fenton reaction [20,21], causing the functional disorder of nucleotides, proteins and other biological molecules through oxidative damage [22]. As a common and important ROS, H_2_O_2_ has been widely used in bacterial inactivation and wound disinfection [23]. However, its sterilization process is rather slow and requires a high concentration of H_2_O_2_ (volume ratio: 0.5–3%, ca. 166–1000 mM) to achieve the desired antibacterial effect. Undoubtedly, this can delay wound healing and even cause damage to normal tissue [24]. Hence, on the premise of achieving wound disinfection and reducing H_2_O_2_ concentration, it is obviously necessary to develop antibacterial materials [25]. In this regard, with the help of the Fenton-like reaction, peroxidase mimics can cause H_2_O_2_ to produce •OH, which exhibits greater toxicity towards bacteria. Therefore, the rational design of a peroxidase mimic as an antibacterial material is an effective measure to improve the antibacterial effect and avoid the side effects caused by a high concentration of H_2_O_2_ [5].

In addition, ascorbic acid (AA) is an important neurochemical substance in living organisms that is often used as an antioxidant in cells and as a protective agent in the nervous system [26]. Abnormal levels of AA in the body can lead to various diseases such as scurvy, mental illness, Alzheimer’s disease, etc. [27]. Evidently, whether the AA content is normal or not in the organism is a significant indicator for the prevention and diagnosis of some diseases. Therefore, it is urgent to develop a simple and high-efficiency AA-detection platform in many fields including the pharmaceutical industry, health monitoring, and so on. As we all know, AA as an antioxidant can inhibit the catalytic oxidation of the chromogenic substrate by peroxidase mimics. Therefore, peroxidase mimics also have great application potential in colorimetric biosensing. However, designing a peroxidase mimic with bifunctional effects that can be used for both broad-spectrum antibacterial applications and AA detection still remains a huge challenge.

Transition-metal sulfides, such as copper sulfide, cobalt sulfide, iron sulfide, etc., are potential peroxidase mimics owing to their adjustable structure/component and diverse morphologies. Especially, copper sulfides can convert H_2_O_2_ to •OH by Fenton-like reactions over a wide pH range. More importantly, the catalytic efficiency of Cu-containing nanomaterials involved in Fenton-like reactions is almost 160-fold higher than that of Fe-based nanozymes in neutral and weakly acidic conditions [2]. Hence, researchers have explored many Cu-containing peroxidase mimics and applied them to the study of colorimetric detection and antibacterial applications. For example, Qiang Bai et al. [28] prepared graphdiyne nanowalls wrapped around a hollow copper sulfide nanocube (CuS@GDY) as a peroxidase mimic, which possessed rapid, efficient, broad-spectrum antibacterial activity against methicillin-resistant *Staphylococcus aureus* and *Escherichia coli*. Additionally, Yuanxiang Xie [29] and his colleagues fabricated the LS-CuS@PVA composite with excellent peroxidase-like performance by incorporating lignin-CuS into poly(vinyl alcohol) (PVP). The antibacterial measurements of the LS-CuS@PVA nanocomposite displayed a high antibacterial rate against *Escherichia coli* and *Staphylococcus aureus* in the presence of H_2_O_2_ under near-infrared light irradiation for 10 min. Therefore, the development of Cu-containing antimicrobials is considered a promising approach to the growing global crisis of antibiotic resistance [30]. In addition, Jing Gao’s group [27] developed a simple method to synthesize the polyacrylonitrile–copper oxide (PAN–CuO) nanoflower with excellent peroxidase-mimicking activity; therefore, they established a colorimetric platform for AA detection.

Although some progress has been made in the research of copper sulfide as a peroxide mimic, few copper-based nanozymes can simultaneously possess broad-spectrum antibacterial properties and colorimetric AA biosensing. Moreover, the current research on peroxidase mimics is mainly focused on single-metal nanozymes because of their relatively clear and simple catalytic mechanism [31]. However, most single-metal nanozymes have low catalytic activity, which lowers their application value. Hence, it is urgent for Cu-based sulfides to further optimize and improve the peroxidase-like activity. Since the catalytic activity of nanozymes is directly related to their composition and structure, designing a unique morphology to increase the exposure of active sites is beneficial to improving their catalytic activity. Additionally, element doping is also an effective method to adjust the composition and electronic structure of nanomaterials. It is reported that bimetallic nanozymes can be synthesized through doping with another metal atom, and the synergistic effect of two metal active centers is expected to improve the catalytic performance [32]. For instance, Chunqiao Jin et al. [3] doped Si into CoO nanorods to improve their peroxidase-mimicking properties, which was much higher than that of pure CoO. They revealed that the enhanced peroxidase-like activity is attributed to the increase in oxygen vacancy. In addition, Le Deng’s group [16] synthesized gold-doped platinum nanodots (AuPtNDs), which exhibited improved peroxidase-like activity that was even higher than horseradish peroxidase. Therefore, the antibacterial performance of the AuPtNDs against both *Escherichia coli* (Gram-negative) and *Staphylococcus aureus* (Gram-positive) was significantly enhanced.

Inspired by the above-mentioned research progress, herein we developed a versatile strategy to synthesize a Ni_0.1_Cu_0.9_S nanoflower through element doping and morphology design. The obtained Ni_0.1_Cu_0.9_S nanoflower was composed of many ultrathin nanosheets, which endowed it with a large surface area in order to expose more active sites and assist the rapid diffusion/penetration of the reaction solution. Additionally, Ni doping brings about a large number of lattice defects, releasing more active sites containing unsaturated dangling bonds. Meanwhile, Ni doping induces the increase in electric dipole and electron transfer, causing Cu and S sites to have partial positive charges, which is beneficial to capturing bacteria and damaging the bacterial cell membrane. As a consequence, the Ni_0.1_Cu_0.9_S nanoflower exhibits prominent peroxidase-like activity, which can efficiently catalyze the decomposition of H_2_O_2_ into more toxic ROS against bacteria and oxidize TMB to generate an obvious blue color. Consequently, the Ni_0.1_Cu_0.9_S nanoflower was used as a broad-spectrum antibacterial agent, which displayed a promising antibacterial property toward Gram-negative *E. coli* and Gram-positive *S. aureus* in the presence of low levels of H_2_O_2_ (0.1 mM). Meanwhile, on the basis of the inhibitory effect induced by AA on the color-rendering process of TMB oxidation, a novel, sensitive and effective Ni_0.1_Cu_0.9_S-based naked-eye biosensor for AA detection was established. In a word, the Ni_0.1_Cu_0.9_S nanoflower as a peroxidase mimic could offer a bifunctional platform for biological assay and antibacterial applications. This work is likely to expand the research value of Cu-containing nanozymes in other biologically related fields.

## 2. Materials and Methods

### 2.1. Chemicals and Materials

Nickel chloride hexahydrate (NiCl_2_·6H_2_O, ≥98.0%), ascorbic acid (AA, AR), and thiourea (CH_4_N_2_S, AR) were purchased from Shanghai Macklin Biochemical Co., Ltd., Shanghai, China. 3,3′,5,5′-tetramethylbenzidine (TMB, ≥98%) and p-benzoquinone (C_6_H_4_O_2_, 99%) were purchased from Shanghai Aladdin Biochemical Technologh Co., Ltd., Shanghai, China. Sulfur sublimed (S, AR), sodium acetate trihydrate (CH_3_COONa·3H_2_O, AR), copper nitrate trihydrate (Cu(NO_3_)_2_·3H_2_O, AR), ethanol (CH_3_CH_2_OH, AR), hydrogen peroxide (30 wt%, H_2_O_2_, AR), and glacial acetic acid (CH_3_COOH, AR) were purchased from Chengdu Cologne Chemicals Co., Ltd., Chengdu, China. l-arginine, l-valine, l-methionine, l-histidine, l-glutamate, glycine, Dl-aspartic acid, l-threonine, l-cysteine and other amino acids were purchased from Sinopharm Chemical Reagents Co., Ltd. (BR), Shanghai, China. Gram-negative *E. coli* (ATCC25922) and Gram-positive *S. aureus* (ATCC29213) were obtained from Shanghai Jiachu Bioengineering Co., Ltd., Shanghai, China. Propidium iodide (PI) and SYTO 9 were obtained from Invitrogen Life Technology Co., Ltd., California, America. Ultra-pure water (18.2 M Ω cm) was used in all solutions and experiments. All chemicals were not further purified. Caution: These reagents are toxic, please use as required.

### 2.2. Synthesis of Ni_0.1_Cu_0.9_S Nanoflower

Ni_0.1_Cu_0.9_S nanoflower was prepared by a one-step hydrothermal method. First, 16 mL anhydrous ethanol were accurately added to the beaker, followed by 0.125 mmol Cu(NO_3_)_2_·3H_2_O, 0.125 mmol NiCl_2_·6H_2_O and 0.5 mmol sulfur powder (Caution: These reagents are toxic, please use as required). Stirring them evenly, afterward, the solution was quickly transferred into a 25 mL Teflon-lined stainless-steel autoclave and maintained at 140 °C for 6 h. Then, the black solid products were collected by centrifuging at 10,000× *g* rpm/min several times. Finally, the Ni_0.1_Cu_0.9_S nanoflower was washed with distilled water and absolute ethanol three times each. After drying overnight in an oven at 60 °C, the Ni_0.1_Cu_0.9_S nanoflower was obtained. The synthesis of pure CuS was similar to the aforementioned method, and the only difference is that no NiCl_2_·6H_2_O was added in the preparation process.

### 2.3. Peroxidase-Like Property of Ni_0.1_Cu_0.9_S Nanoflower

In this study, the peroxidase-like activity of the Ni_0.1_Cu_0.9_S nanoflower was determined by measuring the absorbance of TMB solution in the presence of H_2_O_2_. Briefly, 500 μL Ni_0.1_Cu_0.9_S (10 μg/mL), 500 μL H_2_O_2_ (25 mM) and 500 μL TMB (1.6 mM) were poured into 500 μL HAc-NaAc buffer solution (pH = 5.2) at room temperature. Later, UV–vis spectrophotometry was carried out to monitor the absorbance of the reaction solution at 652 nm. To optimize the reaction conditions, the influences of the pH value, concentration and reaction time on the catalytic activity of the Ni_0.1_Cu_0.9_S nanoflower were also studied.

The steady-state kinetics of the Ni_0.1_Cu_0.9_S nanoflower were studied at room temperature. Firstly, TMB was used as the substrate to detect the affinity between the catalyst and TMB. Then, 500 μL 40 μg/mL Ni_0.1_Cu_0.9_S dispersion, 500 μL 10 mM H_2_O_2_ and 500 μL of different concentrations of TMB (0.25~3.0 mM) were added to the NaAc-HAc buffer solution (0.2 M pH 5.2). Following that, the kinetic spectrum of the reaction system was immediately monitored for the first 600 s and the absorbance of the reaction system at 652 nm was recorded every 1 s. Similarly, H_2_O_2_ was used as the substrate to detect the affinity between the catalyst and H_2_O_2_. More specifically, 500 μL 40 μg/mL Ni_0.1_Cu_0.9_S dispersion, 500 μL 2 mM TMB and 500 μL of different concentrations of H_2_O_2_ (1~30 mM) were added to NaAc-HAc buffer solution (0.2 M pH 5.2). The remaining steps were the same as above. The Michaelis–Menten constant (*K*_m_) was calculated from the following equation:

*V*_0_ = *V*_m_[S]/(*K*_m_ + [S])

where *V*_m_ is the maximum reaction rate, *V*_0_ is the initial rate, [S] refers to the substrate (H_2_O_2_ or TMB) concentration, and *K*_m_ is the substrate concentration when the reaction rate reaches half of the maximum reaction rate.

For the free-radical-capture experiments, 500 μL 40 μg/mL Ni_0.1_Cu_0.9_S dispersion, 250 μL HAc-NaAc buffer solution (0.2 M pH 5.2), 250 μL 1 mM free-radical-trapping agent (p-benzoquinone or thioureas), 500 μL 10 mM H_2_O_2_ and 500 μL 2 mM TMB were mixed and shaken adequately at room temperature. Then, the absorbance of the reaction solution at 652 nm was immediately detected by a UV–vis spectrophotometer. Each group was tested in parallel three times.

### 2.4. Antibacterial Experiments In Vitro

The antibacterial ability of the Ni_0.1_Cu_0.9_S nanoflower was determined by the plate-counting method. Firstly, the bacterial suspension (2 × 10^8^ CFU/mL, 100 μL) was incubated with buffered solution (control), H_2_O_2_ (0.1 mM), Ni_0.1_Cu_0.9_S nanozyme (40 μg/mL), and Ni_0.1_Cu_0.9_S nanozyme (40 μg/mL) + H_2_O_2_ (0.1mM) in a 96-well plate, respectively. After incubation at 37 °C for 18 h, the bacterial suspension was diluted by 1~10^4^ fold, and then 100 μL of the diluted bacterial suspension was spread on the TSA plates. The number of bacterial colonies was counted and recorded after incubation for 16 h at 37 °C. Each group was performed in parallel three times. The antibacterial rate was calculated based on the equation:

Antibacterial rate (%) = 100 − (*N*_t_/*N*_0_) × 100

where *N*_t_ represents the bacterial number of experimental plates, and *N*_0_ represents the bacterial number of blank plates.

To investigate the possibility of sterilization, the solutions prepared in the blank group, the control group and the experimental group were incubated with 10 μL of Dead/Live dye for 15 min, respectively. Propidium iodide (PI) was used to stain dead bacteria with red fluorescence and SYTO 9 was used to stain live bacteria with green fluorescence (Caution: These reagents are toxic, please use as required). Afterwards, the OLYMPUS BX53M fluorescence microscopy (Japan) was used to observe the results.

The minimal inhibitory concentrations (MICs) of Ni_0.1_Cu_0.9_S nanozyme towards *E. coli* and *S. aureus* were determined. For Gram-negative *E. coli*, Ni_0.1_Cu_0.9_S nanozyme solution with different concentrations (0, 0.01, 0.02, 0.04, 0.08, 0.1, 0.2, 0.4, 0.8, 1.6 mg/mL) were added to the bacterial suspensions containing the same number of bacteria. Afterwards, the suspensions were incubated at 37 °C for 24 h. Then, we determined the optical density of bacterial suspensions at 600 nm, and the bacterial suspensions were further diluted and cultured on agar plates for 16 h to count the bacterial colonies. Each group was tested in parallel three times. For Gram-positive *S. aureus*, the same method was carried out.

### 2.5. Detection and Analysis of Ascorbic Acid

Firstly, 500 μL 40 μg/mL Ni_0.1_Cu_0.9_S dispersion, 500 μL 10 mM H_2_O_2_ and 500 μL 2 mM TMB were sequentially poured into 250 μL HAc-NaAc buffer solution (0.2 M pH 5.2). After incubation for 30 min, 250 μL ascorbic acid at different concentrations (0~1.5 mM) was added to the above reaction solution, and then shaken to react for 10 min at room temperature. The absorbance at 652 nm was recorded by a UV–vis spectrophotometer. Each group was tested in parallel three times.

To explore the anti-interference ability of **the** Ni_0.1_Cu_0.9_S nanozyme, 500 μL 40 μg/mL Ni_0.1_Cu_0.9_S dispersion, 250 μL HAc-NaAc buffer solution (0.2 M pH 5.2), 250 μL 10 mM H_2_O_2_, 250 μL 1500 μM ascorbic acid and 250 μL 30 mM interference ions (Zn^2+^, Mg^2+^, Ba^2+^, Ca^2+^, K^+^, Al^3+^, Cd^2+^, Na^+^) were mixed with 500 μL 2 mM TMB, and the total volume of the final system was 2 mL. Following that, the mixture was shaken and placed at room temperature to react for 10 min. Immediately, a UV–vis spectrophotometer was used for spectral detection, and the absorbance value at 652 nm was recorded. For the selectivity measurements of the Ni_0.1_Cu_0.9_S nanoflower, AA was substituted by 250 μL 9 mM amino acids (for instance, l-arginine, l-valine, l-methionine, l-histidine, l-glutamate, glycine, Dl-aspartic acid, l-threonine, l-cysteine, l-tryptophan). Each group was tested in parallel three times.

For the AA detection in real samples, orange juice was diluted 1000-fold and measured without further pretreatment. Firstly, 500 μL 40 μg/mL Ni_0.1_Cu_0.9_S nanozyme, 500 μL 10 mM H_2_O_2_, 500 μL 2 mM TMB and 250 μL diluted orange juice were added to 250 μL HAc-NaAc buffer solution (pH = 5.2). Then, a series of different concentrations of AA solution were also added to the reaction solution according to the standard addition method. Following that, the reaction was conducted at room temperature for 10 min. Finally, the recoveries of AA were calculated on the basis of the linear standard curve. All determinations were performed in triplicate.

In order to test the stability of the Ni_0.1_Cu_0.9_S nanoflower, the above reaction solution was shaken and placed at room temperature for reaction. The absorbance values at 652 nm were recorded by a UV–visible spectrophotometer for 30 consecutive days. In addition, recyclability of the Ni_0.1_Cu_0.9_S nanoflower was evaluated by repeatedly testing the peroxidase-like property of the catalyst cycle by cycle. Considering the mass loss during centrifugation, this measurement was repeated five times to observe the changes.

## 3. Results

### 3.1. Component and Structure Characterization of Ni_0.1_Cu_0.9_S Nanoflower

**Figure 1:**
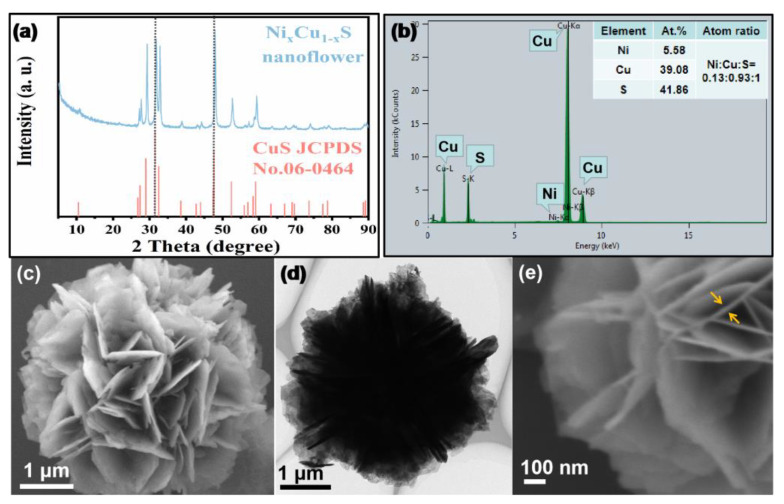
(**a**) The XRD pattern, (**b**) EDS spectrum (the inset shows the atomic percentage), (**c**) low-magnification SEM image, (**d**) low-magnification TEM image and (**e**) high-magnification SEM image of Ni_0.1_Cu_0.9_S nanoflower.

The crystal structure and chemical composition of the as-synthesized material are firstly examined. In Figure 1a, the XRD diffraction peaks can be indexed to the hexagonal CuS phase (06-0464) [33]. No diffraction peaks of Ni species appear, implying that Ni atoms might be doped into the crystal lattice of CuS to form a solid solution. A closer observation reveals there is a slight shift toward high angles in the diffraction peaks, probably caused by the smaller atomic radius of Ni. More-detailed structural information can be collected from the Raman spectrum. According to the above XRD data suggesting that the sample has a hexagonal crystal structure, only S atoms can vibrate in the Raman-active mode [34]. Thus, the Raman spectrum can reflect the polarizability change of S atoms after Ni atoms occupy the Cu sites. As depicted in Figure S1 of Supplementary Materials, the Raman bond at 475 cm^−1^ is indexed to the characteristic stretching vibration of the Cu-S bond in CuS [35]. It is worth noting that the vibration mode of Cu-S in Ni_x_Cu_1−x_S is red-shifted relative to pure CuS. Moreover, the half-peak width increases and the intensity obviously reduces, which indicates there is a smaller polarizability of S atoms after doping with Ni atoms, considering the strong covalent nature of the S atom π-donation to the metal center. This might rationalize that Ni replaces the Cu atom in the Cu-S-Ni configuration, inducing the electron delocalization of S atoms to the neighboring Ni^2+^ sites, and then decreasing the polarizability of S atoms in Ni_x_Cu_1−x_S. The EDS analysis (Figure 1b) confirms that the material is composed of Ni, Cu and S elements with the molar ratio of Ni:Cu:S = 0.13:0.93:1.00, which is in accordance with the ICP-OES result (Table S1). To be clear, there is a large error in the content of the S element measured by ICP-OES, which may be because a large amount of S element is lost in the form of H_2_S during the digestion process. However, this method does not affect the determination of the Ni:Cu atomic ratio (0.1:0.86). These results confirm that the as-prepared sample is the Ni_0.1_Cu_0.9_S nanoflower.

Later, SEM and TEM were carried out to characterize the microstructure and morphology of the Ni_0.1_Cu_0.9_S nanoflower. Figure 1c,d display the Ni_0.1_Cu_0.9_S nanoflower with a diameter of ~4 μm as a loose flower-like ball composed of some uniform nanosheets stacked together. The nanosheet possesses a smooth surface and its thickness is less than 10 nm (Figure 1e). This morphology is able to facilitate the rapid diffusion/penetration of the reaction solution. Meanwhile, it may provide a large surface area in order to expose more active sites. The BET measurement (Figure S2) confirms that the specific surface area is as high as 28.87 m^2^ g^−1^ with the pore size distribution of ~3.84 nm, which is conducive to the improvement of its enzyme-like performance.

**Figure 2:**
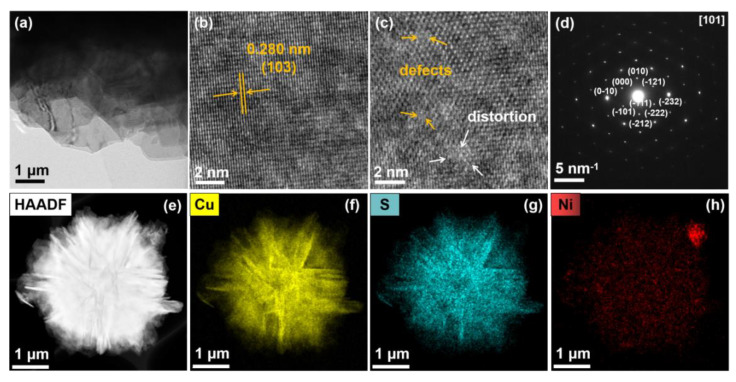
(**a**) TEM image, (**b**) HRTEM image of nanosheets, (**c**) partial enlarged HRTEM image, (**d**) SAED pattern, (**e**) HAADF—STEM image and (**f**–**h**) the EDX elemental—mapping images of Ni_0.1_Cu_0.9_S nanoflower.

Following that, the structure of the Ni_0.1_Cu_0.9_S nanoflower was further studied by HRTEM and selected area electron diffraction (SAED). In Figure 2a, the nanosheet presents many wrinkles, implying the ultrathin feature, and the HRTEM image (Figure 2b) shows a set of distinct lattice fringes. The lattice spacing is determined to be 0.28 nm, corresponding to the (103) facets of hexagonal CuS, which is in agreement with the previously reported literature [36,37]. The zoomed-in image (Figure 2c) shows some defects and lattice distortion, presumably caused by the Ni doping. The typical SAED pattern of the hexagonal structure reveals the single-crystalline feature and can be indexed as the (101) zone axis of CuS, which is in accordance with the HRTEM results. Subsequently, the energy-dispersive X-ray spectroscopy (EDX) coupled with high-angel annular dark-field scanning transmission electron microscopy (HAADF–STEM) was also applied to analyze the element distribution. As shown in Figure 2e–h, it stands to reason that Ni, Cu and S elements are uniformly distributed on the surface of the Ni_0.1_Cu_0.9_S nanoflower, indicating that Ni was homogeneously doped into the CuS nanoflower. Moreover, the EDX analysis further confirms the atomic composition of Ni:Cu:S in the material, which is approximate to the EDS and ICP results.

**Figure 3:**
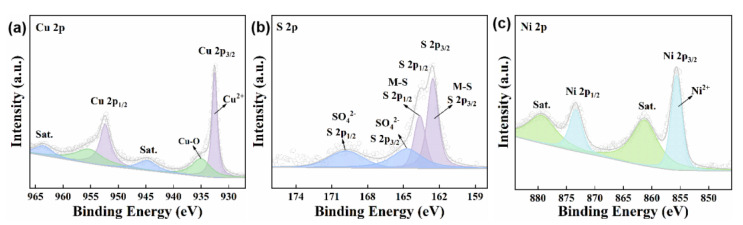
High-resolution XPS spectra of (**a**) Cu 2p, (**b**) S 2p, and (**c**) Ni 2p for Ni_0.1_Cu_0.9_S nanoflower.

In order to gain in-depth insight into the chemical composition and elemental valence state, X-ray photoelectron spectroscopy (XPS) was further conducted with the as-prepared Ni_0.1_Cu_0.9_S nanoflower. The XPS full-survey spectrum in Figure S3a exhibits the characteristic peaks for the Cu, Ni, S, C and O elements, wherein C and O elements might be derived from the surface contaminants and oxidation. This indicates the existence of Cu, Ni and S on the surface of the Ni_0.1_Cu_0.9_S nanoflower without detectable impurities, in good accordance with the EDS results. Afterwards, the binding energies of all spectra were normatively calibrated by the standard C 1s peak (284.8 eV), as shown in Figure S3b. The high-resolution XPS spectrum of the Cu 2p region (Figure 3a) can be deconvoluted into two spin-orbit doublets with two adjacent shake-up satellite peaks. The XPS fitted peaks at ca. 952.6 eV and 932.5 eV with a splitting of 19.9 eV are ascribed to Cu 2p_1/2_ and Cu 2p_3/2_ of Cu^2+^ [35]. Additionally, two deconvoluted shoulder peaks at 955.0 eV (Cu 2p_1/2_) and 934.8 eV (Cu 2p_3/2_) can be assigned to the Cu-O bond, which might come from a small quantity of CuSO_4_ [38]. Impressively, the shake-up satellite peaks situated at 944.7 eV and 963.5 eV manifest the typical feature of Cu^2+^ in CuS materials [39]. For the S 2p spectrum in Figure 3b, the XPS peaks at 163.7 eV (S 2p_1/2_) and 162.6 eV (S 2p_3/2_) with the spin-orbit coupling separation of binding energy (1.1 eV) demonstrate that the S element existed in the form of sulfides [5,33]. As for the shoulder peaks at ca. 169.5 eV and 164.7 eV, they can be indexed to the sulfate species (e.g., CuSO_4_) caused by the oxidation of sulfides in the air [35]. In Figure 3c, the narrow-scan spectrum of Ni 2p exhibits two main peaks at binding energies of 855.6 eV and 873.3 eV. The corresponding satellite peaks at a higher BE location signify the the spin-orbit doublet of 2p orbital for Ni^2+^, due to the formation of strong Ni-S hybridization with charge donation from the Ni 3d to the S 3p orbital [40]. This result implies the successful doping of Ni into CuS nanoflower. More importantly, the binding energy of Cu 2p in the Ni_0.1_Cu_0.9_S nanoflower positively shifts about 0.4 eV compared to pure CuS in the literature, indicating the lower electron density of Cu 2p along with the doping of Ni. Owing to the higher electronegativity of Ni (*χ* = 1.91), Ni doping results in the increase in the electric dipole and the transfer of more electrons from Cu (*χ* = 1.90) to other atoms [41]. Interestingly, the electrons do not transfer to S atoms, because the XPS peaks of S 2p also shift towards a higher binding energy, which is consistent with the Raman results. These observations illustrate that there may be many cation vacancies. Additionally, the higher binding energy results in the lower electron density, endowing the Cu and S sites with partial positive charges and excellent electron-receiving ability, which is beneficial to exerting its peroxidase-like activity [42]. In terms of antibacterial effect, the Cu and S sites with partial positive charges will cause damage to the bacterial cell membrane [43], which is expected to improve its antibacterial efficiency. Additionally, the surface compositional analysis confirms that the surficial atomic ratio of Ni:Cu:S is determined to be 0.11:0.92:1.00, which is in excellent accord with the ICP-OES result. Taken together, we can conclude the successful formation of the Ni-doped CuS nanoflower (Ni_0.1_Cu_0.9_S nanoflower).

### 3.2. Peroxidase-Like Property of Ni_0.1_Cu_0.9_S Nanoflower

**Figure 4:**
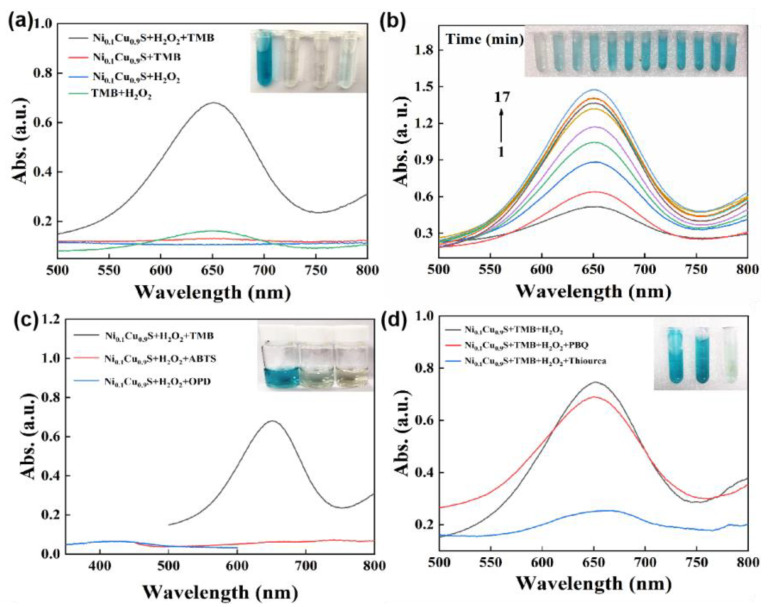
(**a**) The UV–vis absorption spectra of various reaction systems in acetate buffer solution (pH = 5.2) recorded at 10 min. (**b**) The UV–vis absorption spectra over time in the reaction system of Ni_0.1_Cu_0.9_S + H_2_O_2_ + TMB. (**c**) The UV–vis absorption spectra of Ni_0.1_Cu_0.9_S + H_2_O_2_ + different substrate (TMB, OPD or ABTS). (**d**) The UV–vis absorption spectra of Ni_0.1_Cu_0.9_S + H_2_O_2_ + TMB system in the absence or presence of PBQ or thiourea. The insets are their corresponding photographs.

Considering that copper ions are the catalytic active centers of many natural enzymes, some copper-containing compounds can produce ROS through Fenton-like reactions similar to peroxidase, which endows them with potential for bio-applications, such as antibacterial effects, biomolecular assays, and so on. Therefore, once the Ni_0.1_Cu_0.9_S nanoflower was synthesized, we evaluated its peroxidase-like properties by monitoring the catalytic oxidation reaction of 3,3′,5,5′-tetramethylbenzidine (TMB) through a colorimetric method and UV–vis measurement. As depicted in Figure 4a, the Ni_0.1_Cu_0.9_S nanozyme could efficiently catalyze the decomposition of H_2_O_2_ into ROS and oxidize TMB to generate an obvious blue color with a maximum absorbance at 652 nm. The peak was deemed to be a charge-transfer complex with a radical cation (TMB^+^), implying the formation of ox-TMB [44]. However, the Ni_0.1_Cu_0.9_S nanozyme or H_2_O_2_ alone could not produce a similar phenomenon, indicating the peroxidase-like activity of the Ni_0.1_Cu_0.9_S nanoflower. Additionally, the time-course profile in Figure 4b reveals that as the reaction continues, the intensity of the absorbance increases rapidly, accompanied by a darker color in the reaction solution, which proves a fast reaction rate. To distinguish the specificity of the Ni_0.1_Cu_0.9_S nanoflower towards chromogenic substrates, the o-phenylenediamine (OPD) and 2-Azinobis-(3-ethylbenzthiazoline- 6-sulphonate) (ABTS) were also selected as substrates for comparison. By visual inspection and spectral measurements (Figure 4c), neither OPD nor ABTs could be catalyzed by the Ni_0.1_Cu_0.9_S nanozyme to produce the characteristic color and absorbance. This may be due to the locally unbalanced Coulomb interaction resulting from the replacement of Cu atoms by the more electronegative Ni atoms, which makes the catalyst more inclined to bind to the positively charged TMB substrate, rather than the negative ABTs and electrically neutral OPD. Therefore, the Ni_0.1_Cu_0.9_S nanoflower possesses a certain degree of specificity towards chromogenic substrates. In order to determine the possible ROS, radical-quenching experiments were conducted to explicate the predominant species generated during H_2_O_2_ decomposition. In Figure 4d, thiourea and p-benzoquinone (PBQ) as the free-radical quenchers of hydroxyl radical (•OH) and superoxide anion (O_2_^•−^) [45], respectively, were added to the reaction system of Ni_0.1_Cu_0.9_S + TMB + H_2_O_2_. Evidently, the intensity of absorbance drops dramatically when the thiourea is added to the reaction system. Additionally, PBQ also exhibits a slight inhibition of the catalytic activity of the Ni_0.1_Cu_0.9_S nanoflower. The above results confirm that the hydroxyl radical (•OH) is the predominant ROS, accompanied by a small dose of superoxide anion (O_2_^•−^) in the peroxide-mimicking catalytic process of the Ni_0.1_Cu_0.9_S nanoflower. Therefore, it can be described that the excellent peroxidase-like activity of the Ni_0.1_Cu_0.9_S nanoflower stems from the ROS (•OH and O_2_^•−^) generated with the assistance of H_2_O_2_, laying the foundation for further biomedical applications in the fields of antibacterial action and colorimetric detection.

To improve the catalytic activity of the Ni_0.1_Cu_0.9_S nanoflower, multiple conditions, such as pH value, reaction time, TMB, H_2_O_2_ and catalyst concentrations, were thoroughly explored. Similar to a natural enzyme, the catalytic activity of a nanozyme is also affected by pH value. Consequently, we firstly researched the dependence of the peroxidase-like property on pH value. As displayed in Figure S4a,b, it can be seen that the Ni_0.1_Cu_0.9_S nanoflower shows an outstanding peroxidase-like activity over a wide pH range (3.2~7.2) and reaches optimal conditions at pH = 5.2. As is well known, the catalytic activity at physiological pH values is a distinctly important indicator for evaluating the capabilities for biomedical research of enzyme mimics. The Ni_0.1_Cu_0.9_S nanoflower, here, revealed prominent peroxidase-like activity over the physiologically important pH range of 4.0–7.4, especially close to the weak acid pH (~6.2) of wounds, which satisfies a prerequisite for its bio-application at physiological conditions. Additionally, in Figure S4c,d, the absorbance at 652 nm increases gradually over time and eventually stabilizes after 10 min, that is to say that the Ni_0.1_Cu_0.9_S nanoflower as a peroxidase mimicase can complete the catalytic oxidation of TMB within 10 min. Accordingly, the optimal response time was set as 10 min. Following that, the influences of TMB, H_2_O_2_ and catalyst concentrations on peroxidase-like activity were subsequently explored. In Figure S4e–j, with the increase in concentration, the absorbance increases until saturation. To guarantee the accuracy, the optimized concentration of TMB, H_2_O_2_ and the catalyst was set as 2 mM, 10 mM and 40 μg/mL, respectively.

For an ideal nanozyme, the stability and reusability are important references to measure its practical value. Figure S5 shows that the peroxidase-like activity remains at 88.8% within a 30-day storage period in aqueous solution and the absorbance keeps basically steady for five cycles, except for the attenuation due to the mass loss during centrifugal operation after each cycle. In addition, the SEM image (Figure S6) after the stability test demonstrates an almost unchanged morphology. All the evidence concludes that the Ni_0.1_Cu_0.9_S nanoflower possesses remarkable stability and reusability, indicating the prospect of practical applications in the field of catalysis.

To gain a better understanding of the peroxidase-like catalytic process of the Ni_0.1_Cu_0.9_S nanoflower, a steady-state kinetics analysis was carried out. Typical Michaelis–Menten curves (Figure S7) were plotted over a certain range of TMB and H_2_O_2_ concentrations. Obviously, the initial velocity rapidly increases with the increase in TMB or H_2_O_2_ concentration. The Michaelis–Menten constant (*K_m_*) and maximal reaction velocity (*V_max_*) were calculated from the Lineweaver–Burk double-reciprocal plots and the results are listed in Table S2. *K_m_* has been identified as an indicator of a catalyst’s affinity to a substrate. A lower *K_m_* value reflects a stronger affinity and vice versa [27]. As listed in Table S2, the *K_m_* value of the Ni_0.1_Cu_0.9_S nanoflower with H_2_O_2_ as a substrate was determined to be 3.698 mM, close to that of HRP (3.7 mM) reported in the previous literature [46], implying a high affinity between the Ni_0.1_Cu_0.9_S catalyst and H_2_O_2_. Likewise, the *K_m_* value (0.359 mM) of the Ni_0.1_Cu_0.9_S nanoflower with TMB as a substrate is even lower than that of HRP (0.43 mM) [47]. Additionally, *V_max_* represents the catalytic ability [11]. Table S2 displays the *V_max_* values with TMB and H_2_O_2_ as 5.60 × 10^−8^ and 5.604 × 10^−8^ M/s, respectively, which are markedly higher than those of other peroxidase mimicases reported in the previous literature, demonstrating a higher catalytic efficiency. The lower *K_m_* and higher *V_max_* values make the Ni_0.1_Cu_0.9_S nanoflower a desirable peroxidase mimicase with satisfying catalytic performances.

### 3.3. Antibacterial Activity Evaluation

**Figure 5:**
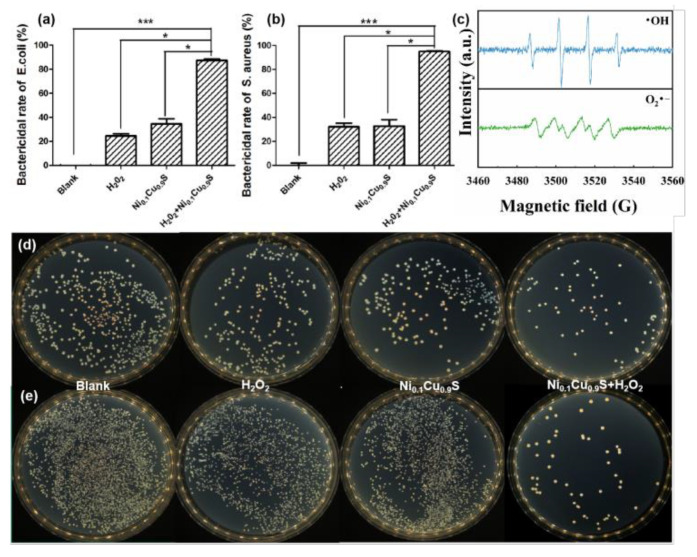
The bactericidal rate of Ni_0.1_Cu_0.9_S nanozyme against (**a**) Gram-negative *E. coli* and (**b**) Gram-positive *S. aureus*. Data are presented as the mean ± SD (*n* = 3). * means *p* < 0.05, *** means *p* < 0.001. (**c**) EPR spectra of superoxide anion and hydroxyl radical in the reaction system. The colony plating images of different systems against (**d**) *E. coli* and (**e**) *S. aureus*.

For antibacterial application, the •OH species (2.8 V) has higher antibacterial ability than H_2_O_2_ (1.8 V) in view of the higher oxidation potential [30,47]. Additionally, the excellent peroxidase-like property of the Ni_0.1_Cu_0.9_S nanoflower can convert H_2_O_2_ into •OH; therefore, the antibacterial system with the Ni_0.1_Cu_0.9_S nanozyme was designed. Two strains associated with medical infections, including Gram-negative *E. coli* and Gram-positive *S. aureus*, were applied for the antibacterial measurements (Figure 5), and the results of the antibacterial evaluation of the Ni_0.1_Cu_0.9_S nanozyme were collected by the plate-counting method in terms of colony formation. Prior to this, we studied the trapping ability of the Ni_0.1_Cu_0.9_S nanozyme for bacteria. As displayed in Figure S8, the Ni_0.1_Cu_0.9_S nanozyme is able to capture about 59.2% *E. coli* and 90.4% *S. aureus* through the electrostatic interaction between positively charged sites and negatively charged bacterial cells. This excellent capture ability is beneficial to limiting the damage of bacteria to ROS within a certain range, and effectively enhances the bacteriostatic efficiency of •OH. Figure 5a,b depict the antibacterial performance of the Ni_0.1_Cu_0.9_S nanozyme for Gram-negative *E. coli* and Gram-positive *S. aureus*, respectively. The antibacterial photos (Figure 5d,e) of the culture plates vividly visualize the survival of bacteria, in which the small white dots reflect the survival colonies. Evidently, the control plates in blank groups display dense colonies of two bacteria, implying their robust growth in the test system. Additionally, in order to avoid the toxicity from high-concentration H_2_O_2_ (0.5–3 wt%) for clinical use, the test systems with biologically relevant levels of H_2_O_2_ concentration were fixed at 0.1 mM, which is very weak against *E. coli* and *S. aureus*. However, both bacteria can hardly survive once40 μg/mL of the Ni_0.1_Cu_0.9_S nanozyme is added to the measurement system for 16 h, initially confirming that the Ni_0.1_Cu_0.9_S peroxidase mimic possesses an antimicrobial effect on the selected bacteria with the assistance of low-dose H_2_O_2_.

It is worth mentioning that the Ni_0.1_Cu_0.9_S nanozyme cannot produce ROS to oxidize TMB without the assistance of H_2_O_2_ as depicted in Figure 4a. However, Figure 5a,b display that the Ni_0.1_Cu_0.9_S nanozyme alone has a certain degree of bactericidal ability. This may be due to the dissociation of trace Cu^2+^ ions, which can penetrate the membrane and denature the DNA/RNA by chelating. This is a very common antibacterial mechanism in metal-based nanozymes [30,48,49]. Therefore, we speculate that the antibacterial mechanism of the Ni_0.1_Cu_0.9_S nanozyme is due to its dual action, that is to say, the ROS storm stemmed from POD-like activity and the chelation derived from dissociated Cu^2+^ ions. In order to confirm the mechanism, the electron paramagnetic resonance (EPR) spectrum was used to provide more direct evidence. 5,5-dimethyl-1-pyridine N-oxide (DMPO) as a spin-capture reagent could bond with oxygen-centered free radicals, such as •OH and O_2_^•−^, to generate the more-stable free-radical adducts [50]. Consequently, the production of •OH and O_2_^•−^ could be monitored by EPR by incorporating DMPO. As shown in Figure 5c, an intense four-line characteristic signal (1:2:2:1) manifests the presence of the DMPO-•OH adduct [51], and a weak signal of six characteristic peaks indicates the DMPO-O_2_^•−^ adduct [52]. These results disclose the production of •OH and O_2_^•−^ in the catalytic reaction solution. Additionally, we further measured the concentration of released Cu^2+^ ions in the reaction system by ICP-OES technology. As shown in Table S3 and Figure S9, the degradation rate is rapid during the first 10 h and then slows. Finally, the concentration of Cu^2+^ ions in the reaction solution gradually remains constant after 25 h. Afterwards, we further evaluated the antibacterial effect of the Cu^2+^-containing supernatant solution towards *E. coli* and *S. aureus* without the assistance of H_2_O_2_. As depicted in Figure S10, the antibacterial efficiency of the supernatant solution slightly enhances with the increase in the concentration of Cu^2+^ ions, illustrating the slight contribution of Cu^2+^ ions to the antibacterial effect. In view of the above-mentioned facts, these results verify our hypothesis that both ROS and degraded Cu^2+^ ions play roles in the antibacterial mechanism of the Ni_0.1_Cu_0.9_S nanozyme.

**Figure 6:**
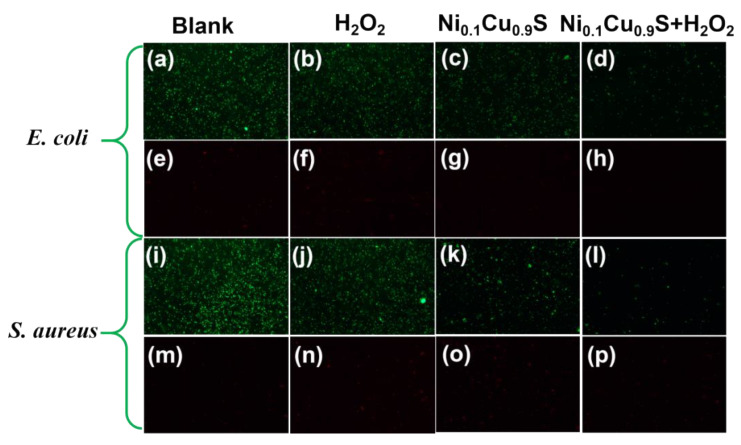
Live-dead fluorescence images of (**a**–**h**) *E. coli* and (**i**–**p**) *S. aureus* colonies, where viable cells were stained green with SYTO 9 and dead cells were stained red with propidium iodide. ((**a**,**e**,**i**,**m**): blank; (**b**,**f**,**j**,**n**): H_2_O_2_-only; (**c**,**g**,**k**,**o**): Ni_0.1_Cu_0.9_S-only; (**d**,**h**,**l**,**p**): Ni_0.1_Cu_0.9_S + H_2_O_2_).

In addition, fluorescence-based Live/Dead bacterial cell staining assays were further conducted to confirm the antibacterial property of the Ni_0.1_Cu_0.9_S nanozyme. SYTO 9 and propidium iodide (PI) were used as the probes of live bacteria and dead bacteria, respectively, because membrane-permeable SYTO 9 can only be marked by green fluorescence in live bacteria and membrane-impermeant PI can only be labeled with dead bacteria with red fluorescence through damaged bacteria membranes [16]. As evidenced in Figure 6, the lack of a red fluorescent signal of the control groups with buffer solution alone, H_2_O_2_ alone and nanozyme alone indicates no obvious antibacterial property. Whereas, an obvious decrease in green fluorescence signal and the dominant red fluorescence signal were observed during simultaneous treatment with both the Ni_0.1_Cu_0.9_S nanozyme and H_2_O_2_ in the buffer solution, manifesting a dramatic increase in dead bacteria. The above results disclose that the Ni_0.1_Cu_0.9_S nanozyme possesses prominent antibacterial activity toward Gram-negative *E. coli* and Gram-positive *S. aureus* when assisted with a low dose of H_2_O_2_.

**Figure 7:**
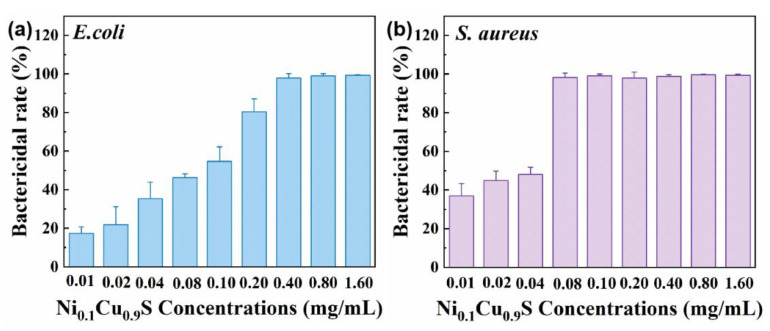
Bactericidal rate of reaction systems against (**a**) Gram-negative *E. coli* and (**b**) Gram-positive *S. aureus*, respectively, treated with different concentrations of Ni_0.1_Cu_0.9_S nanoflower.

Afterwards, the antibacterial effectiveness was also tested by the relationship between the number of *E. coli* and *S. aureus* colonies and the concentration of the Ni_0.1_Cu_0.9_S nanozyme when about 10^8^ CFU of bacteria are applied to the LB plates. Then, we further tested the MIC, which is defined as the lowest concentration of antibacterial agents in solution that completely prevents the growth of bacteria in standard incubation conditions. Clearly, the antibacterial rates of the Ni_0.1_Cu_0.9_S nanozyme towards the two bacteria show a strong dependence on the concentration. As expected, with other conditions being equal, the antibacterial rate raises with the increase in Ni_0.1_Cu_0.9_S concentration (in Figure 7). More importantly, when the concentration of the Ni_0.1_Cu_0.9_S nanozyme reaches 0.4 mg/mL, the antibacterial rate toward *E. coli* is almost 100% and remains fixed when continuing to add more antibacterial materials. As for the antibacterial rates toward *S. aureus*, the Ni_0.1_Cu_0.9_S nanozyme exhibits a maximum killing efficacy almost of 98.2% at the concentration of 0.08 mg/mL. Compared with the blank group, no sight of bacteria on the dishes is found when treated with the Ni_0.1_Cu_0.9_S nanozyme in the presence of H_2_O_2_ (0.1 mM). The observation suggests that the Ni_0.1_Cu_0.9_S nanoflower possesses an excellent broad-spectrum antibacterial property, as well as dose-dependent antibacterial efficacy, which has potential applications in the biomedical field.

The above excellent antibacterial performance is attributed to the following structural advantages: (1) the nanosheet-assembled nanoflower-like morphology provides a large surface area and porous structure, which is conducive to enhancing the contact between substrate and nanozyme, while accelerating the diffusion and infiltration of the reaction solution; (2) Ni doping brings about a large number of lattice defects (e.g., cation vacancies and distortions), releasing more active sites with unsaturated dangling bonds; (3) After doping Ni atoms, the electrons of S atoms are delocalized to the adjacent Ni site to some extent, reducing the polarization of S atoms. Moreover, the Ni doping induces the increase in electric dipole and the transfer of more electrons from Cu to Ni sites. These endow Cu and S sites with partial positive charges, which are beneficial to capturing bacteria and damage the bacterial cell membrane. All the above merits endow the Ni_0.1_Cu_0.9_S nanoflower with satisfying antibacterial activity.

### 3.4. Determination and Colorimetric Assay of Ascorbic Acid

**Figure 8:**
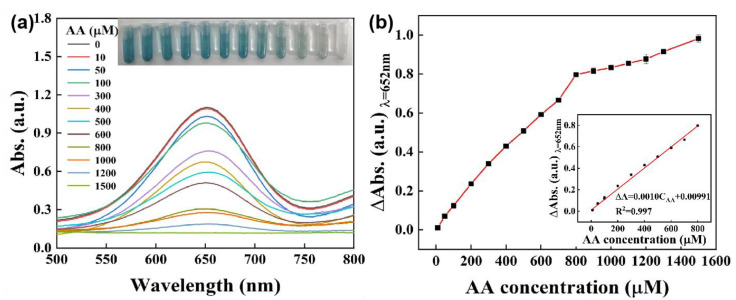
(**a**) The UV–vis absorption spectra of the reaction system of Ni_0.1_Cu_0.9_S + H_2_O_2_ + TMB in the presence of AA with varied concentrations. (The inset displays the color changes of reaction solution with the increasing AA concentration). (**b**) Plots of the absorbance difference (∆A) at 652 nm versus the AA concentration. Inset is the linear calibration plot corresponding to absorbance against the concentration of AA. The error bars represent the standard deviation values of three measurements.

Ascorbic acid (AA) is an important nutrient needed in the metabolic process of human. Although it is well known as an antioxidant that can eliminate free radicals, excessive and inadequate amounts of AA will inevitably lead to various diseases, such as scurvy, cancer, Alzheimer’s disease and kinds of infections [53]. Therefore, it is of great physiological and pathological significance to develop a rapid and effective method to detect AA. Based on the peroxidase-like activity of the Ni_0.1_Cu_0.9_S nanoflower and the inoxidizability of AA, a colorimetric technique for the detection of AA by the unaided eye was designed by the specific inhibition effect toward the catalytic oxidation of colorless TMB into blue ox-TMB. The UV–vis spectrum and color response of the Ni_0.1_Cu_0.9_S + H_2_O_2_ + TMB system were recorded with the addition of different concentrations of AA. As demonstrated in Figure 8a, the absorbance at 652 nm gradually decreases with the increase in AA concentrations from 0 to 1500 μM, along with the fading of the solution color from blue to colorless. Noteworthily, there is a highly linear relationship between AA concentration and the absorbance difference (∆A) (∆A = A_0_ − A, A_0_ represents the absorbance intensity in the absence of AA, A stands for the absorbance intensity in the presence of AA at 652 nm), as exhibited in Figure 8b. In the range of 10 μM–800 μM for AA concentration, the linear regression equation is as follows: ∆A = 0.00076C_AA_ + 0.1099 (R^2^ = 0.997). It can be seen that the absorption intensity of the Ni_0.1_Cu_0.9_S-based colorimetric biosensor is very sensitive to AA concentration. Using a signal-to-noise ratio of 3 (3*σ*/*k*), wherein *σ* and *k* are the standard deviation of blank sample and the slope of the linear fitting curve, respectively, the limit of detection (LOD) for AA is calculated to be as low as 0.84 μM. Table S4 summarizes the detection performance of other materials reported in the previous literature. Obviously, the colorimetric sensor based on the Ni_0.1_Cu_0.9_S nanozyme is more sensitive and reliable compared with the other peroxidase-mimic-based detection platforms for AA listed in Table S4. Therefore, the peroxidase-like property of the Ni_0.1_Cu_0.9_S nanoflower can be successfully developed as a naked-eye colorimetric method for the detection of AA with a good linear relationship and a low detection limit.

**Figure 9:**
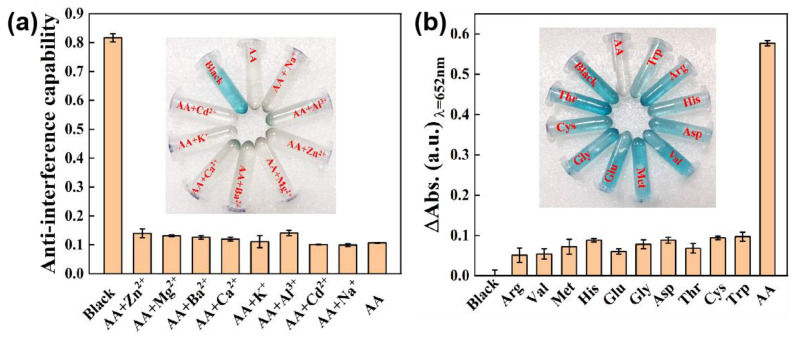
(**a**) Anti-interference ability of the detection platform for AA assay towards coexisting interference substances including Zn^2+^, Mg^2+^, Ba^2+^, Ca^2+^, K^+^, Al^3+^, Cd^2+^ and Na^+^. (**b**) Selectivity of the detection platform for AA assay. From left to right: Blank, l-arginine, l-valine, l-methionine, l-histidine, l-glutamate, glycine, Dl-aspartic acid, l-threonine, l-cysteine, l-tryptophan and ascorbic acid. The error bars represent the standard deviation values of three measurements. The insets are corresponding photographs of color changes.

There is no doubt that selectivity and anti-interference performance are the significant indicators of the biosensor toward AA detection. Hence, the selectivity and anti-interference performance were investigated by replacing AA with other amino acids, such as l-arginine, l-valine, l-methionine, l-histidine, l-glutamate, glycine, Dl-aspartic acid, l-threonine, l-cysteine and l-tryptophan, or by adding interfering ions to measurement systems, for instance, Zn^2+^, Mg^2+^, Ba^2+^, Ca^2+^, K^+^, Al^3+^, Cd^2+^ and Na^+^. The concentrations of the biological molecules were 6-fold greater than AA, and the concentrations of the interfering ions were more than 20 times that of AA. By monitoring the absorbance difference (∆A) of the test systems containing AA and other interfering ions (Figure 9a), it can be concluded there is a negligible effect on the detection of AA, manifesting the outstanding anti-interference ability of this bioassay. What is more, a careful analysis of Figure 9b reveals that the absorbance dramatically drops with the blue fading from the reaction solution only in the presence of AA, while this phenomenon disappears by replacing AA with other amino acids. This result discloses the excellent specificity toward AA detection. In a word, none of the biological substances or interfering ions in this test can cause interference with AA detection at physiological levels, ensuring the selectivity of this colorimetric method. Obviously, the sensing platform possesses prominent anti-interference ability, sensitivity and a quick response to directly detect AA. In order to prove the application prospect of this biosensing platform in real samples, we conducted this method to measure AA concentration in orange juice. The recovery experiments were executed by using the standard addition method, where different concentrations of AA were added to dilute orange juice samples for analysis. The results are listed Table S5. It can be seen that the recovery values are 96.87~105.11% with relative standard deviations (RSD) of 0.62~3.92%. The satisfactory recovery and accuracy illustrate that the biosensing platform possesses a great potential for AA detection in real samples.

## 4. Discussion

In general, we have designed a simple yet versatile method to synthesize the Ni_0.1_Cu_0.9_S nanoflower, which is composed of a large number of ultrathin nanosheets. The nanosheet-assembled nanoflower-like morphology offers a large specific surface area in order to expose more active sites and facilitates the rapid diffusion/penetration of the reaction solution. Additionally, Ni doping brings about a large number of lattice defects, releasing more active sites containing unsaturated dangling bonds. Meanwhile, Ni doping induces the increase in electric dipole and electron transfer, endowing Cu and S sites with partial positive charges, which are beneficial to capturing bacteria and damaging the bacterial cell membrane. By the regulating strategies of Ni doping and morphology design, the prepared Ni_0.1_Cu_0.9_S nanoflower possesses excellent peroxidase-mimic catalytic performance. Combined with the low possibility of bacterial drug resistance to copper-based antibacterial agents, the Ni_0.1_Cu_0.9_S nanoflower exhibits improved broad-spectrum antibacterial activity against Gram-negative *E. coli* and Gram-positive *S. aureus* through the rapid denaturation of bacterial colonies induced by ROS and degraded Cu^2+^ ions at relatively low concentrations of H_2_O_2_. Additionally, the Ni_0.1_Cu_0.9_S nanoflower as an excellent peroxidase mimic could covert colorless TMB to blue ox-TMB with the assistance of H_2_O_2_. Considering the inhibition effect of the antioxidant AA on the peroxidase-like activity of the Ni_0.1_Cu_0.9_S nanoflower, a facile and sensitive colorimetric biosensing method for AA is established. As expected, this analysis assay reveals an excellent response to AA detection with good linearity and an LOD as low as 0.84 μM. In brief, the Ni_0.1_Cu_0.9_S nanoflower as a peroxidase-like catalyst can not only provide a reliable sensing platform for AA detection, but can also kill infection-associated bacteria and avoid the toxicity of H_2_O_2_ as a broad-spectrum antibacterial agent. Consequently, this work is meaningful for exploring the multi-applications of copper-containing nanomaterials in the fields of biomedicine, biosensing and biocatalysis.

## Data Availability

The data presented in this work are available from the corresponding author upon reasonable request.

## Supplementary Materials

The following supporting information can be downloaded at: https://www.mdpi.com/article/10.3390/bios12100874/s1. Figure S1. The Raman spectra of Ni_0.1_Cu_0.9_S nanoflower and pure CuS; Figure S2. N_2_ adsorption–desorption isotherm of the Ni_0.1_Cu_0.9_S nanoflower. The inset is the corresponding pore size distribution curve; Figure S3. (a) The XPS survey scan of the Ni_0.1_Cu_0.9_S nanoflower. (b) High-resolution XPS spectrum of C 1s for Ni_0.1_Cu_0.9_S nanoflower; Figure S4. The influences of (a–b) pH value, (c–d) reaction time, (e–f) TMB concentration, (g–h) H_2_O_2_ concentration and (i–j) catalyst concentration on the peroxidase-like activity of the Ni_0.1_Cu_0.9_S nanozyme. The error bars represent the standard deviation values of three measurements; Figure S5. (a) Long-term stability of Ni_0.1_Cu_0.9_S nanozyme for peroxidase-like activity. (b) The UV–vis absorption value of relative catalytic activity for five cyclic experiments; Figure S6. The SEM image of Ni_0.1_Cu_0.9_S nanozyme after the stability test; Figure S7. (a) and (b) the Michaelis–Menten curve for H_2_O_2_ and TMB, respectively. (c) and (d) the Lineweaver–Burk plot for determination of kinetic constant of Ni_0.1_Cu_0.9_S nanoflower for H_2_O_2_ and TMB, respectively; Figure S8. Relative OD_600_ values of Ni_0.1_Cu_0.9_S nanozyme towards *E. coli* and *S. aureus*; Figure S9. The release curve of time-dependent Cu^2+^ of Ni_0.1_Cu_0.9_S nanozyme in test system plotted with data obtained by ICP; Figure S10. Survival rates of *E. coli* and *S. aureus* treated with Cu^2+^ supernatant samples. Table S1. Summary of ICP-OES results for Ni_0.1_Cu_0.9_S nanoflower; Table S2. Comparison of the K_m_ and V_max_ values for Ni_0.1_Cu_0.9_S nanoflower with those of other peroxidase mimicase; Table S3. The ICP-OES results of cumulative Cu^2+^ release from Ni_0.1_Cu_0.9_S nanozyme in test system; Table S4. Comparison of different sensors for AA detection; Table S5. Determination of the amounts of AA in real samples (n=3) [54,55,56,57,58,59,60,61,62,63,64,65,66,67,68,69,70,71].
