# Supplementary material for: Exploring the Antibacterial and Biosensing Applications of Peroxidase-Mimetic Ni0.1Cu0.9S Nanoflower
Source: Biosensors (Basel). 2022 Oct 15;12(10):874. doi: 10.3390/bios12100874 (PMC9599305; doi:10.3390/bios12100874)
Supplement: Supplementary file 1 [file biosensors-12-00874-s001.zip › biosensors-1953384-supplementary.pdf]

Supplementary Materials:

Exploring the antibacterial and biosensing applications of peroxidase-mimetic $Ni_{0.1}Cu_{0.9}S$ nanoflower

Li Liu,[1†] Yayu Lai,[2†] Jinming Cao,[1] Yu Peng,[1] Tian Tian[*1] and Wensheng Fu[*1]

[1] Chongqing Key Laboratory of Green Synthesis and Applications, College of Chemistry, Chongqing Normal University, Chongqing 401331, China

[2] The Department of General Practice, the 958th hospital of Chinese People's Liberation Army, Chongqing 400000, China

* Correspondence: tthy6080@163.com (T.T.); fuwensheng@cqnu.edu.cn (W.F.)

† These authors contributed equally to this work.

Keywords: peroxidase mimicase; reactive oxygen species; antibacterial application; biosensing; copper-containing nanozymes

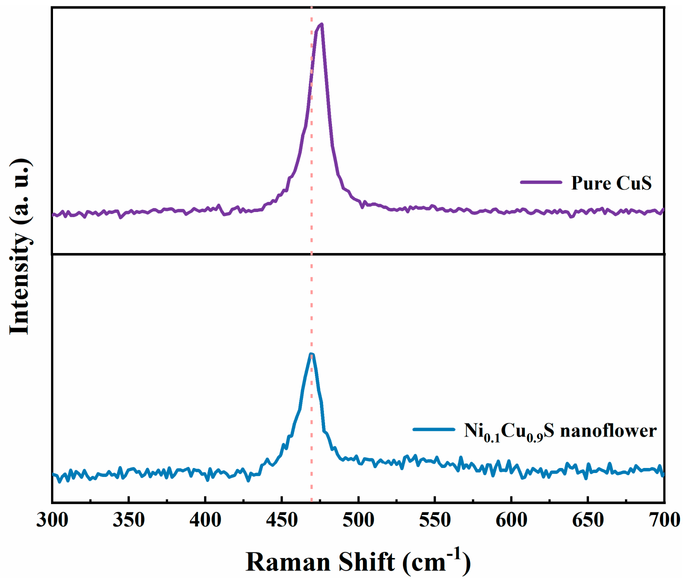

Figure S1. The Raman spectra of $Ni_{0.1}Cu_{0.9}S$ nanoflower and pure CuS.

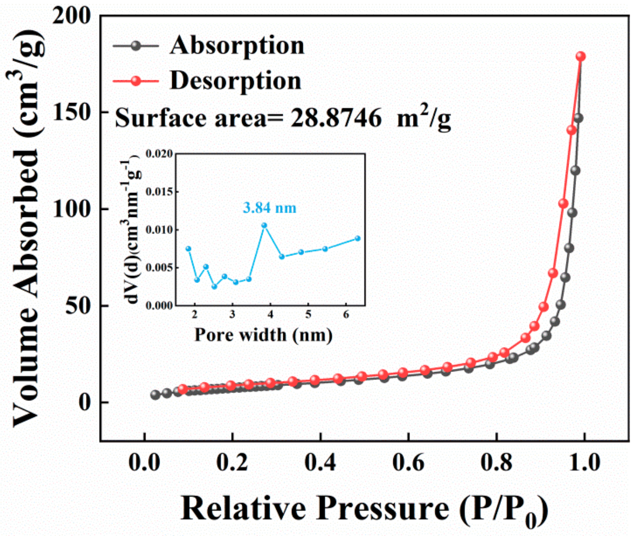

Figure S2. N_2 adsorption-desorption isotherm of the $Ni_{0.1}Cu_{0.9}S$ nanoflower. The inset is the corresponding pore size distribution curve.

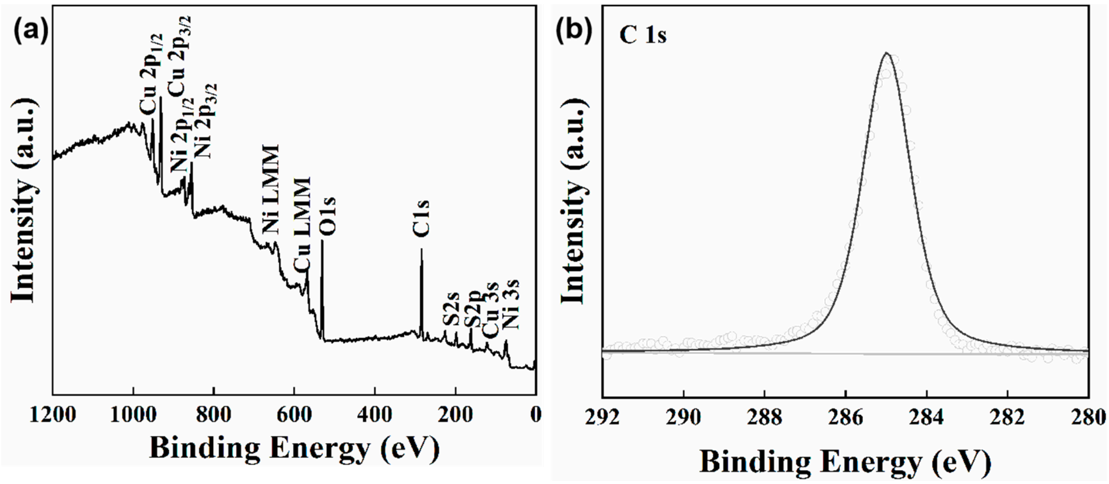

Figure S3. (a) The XPS survey scan of the $Ni_{0.1}Cu_{0.9}S$ nanoflower. (b) High-resolution XPS spectrum of C 1s for $Ni_{0.1}Cu_{0.9}S$ nanoflower.

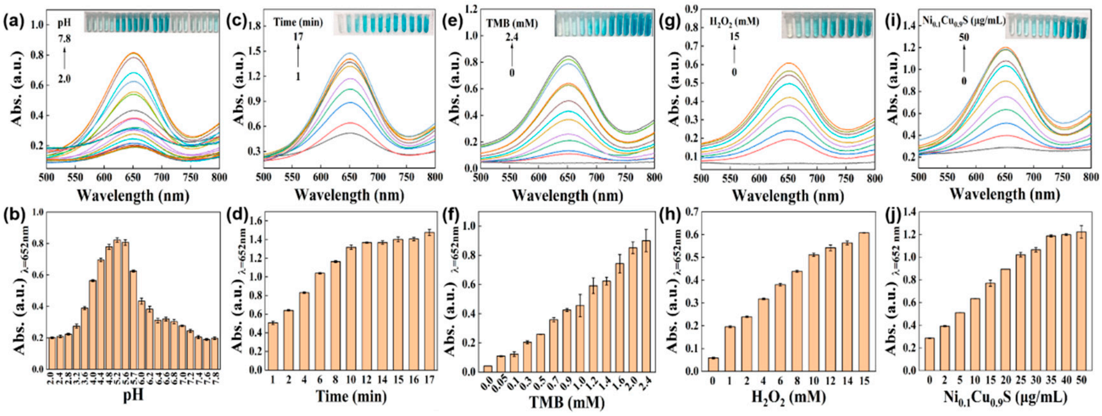

Figure S4. The influences of (a-b) pH value, (c-d) reaction time, (e-f) TMB concentration, (g-h) H_2O_2 concentration and (i-j) catalyst concentration on the peroxidase-like activity of the $Ni_{0.1}Cu_{0.9}S$ nanozyme. The error bars represent the standard deviation values of three measurements.

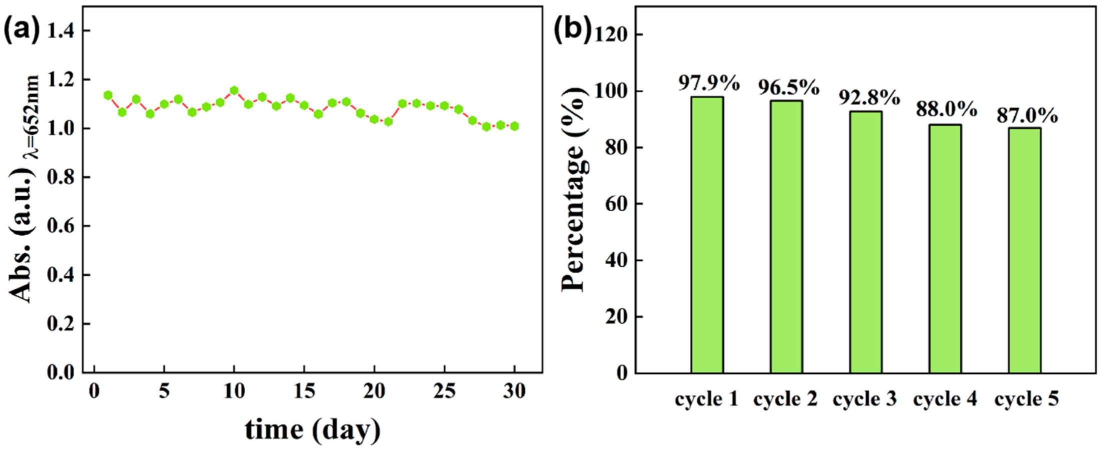

Figure S5. (a) Long-term stability of $Ni_{0.1}Cu_{0.9}S$ nanozyme for peroxidase-like activity. (b) The UV-vis absorption value of relative catalytic activity for five cyclic experiments.

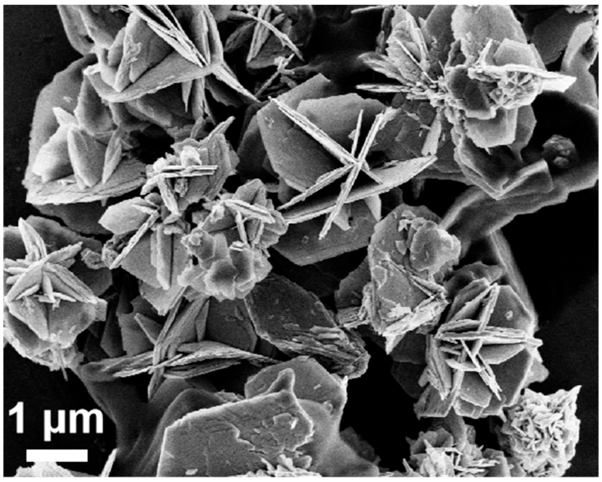

Figure S6. The SEM image of $Ni_{0.1}Cu_{0.9}S$ nanozyme after the stability test.

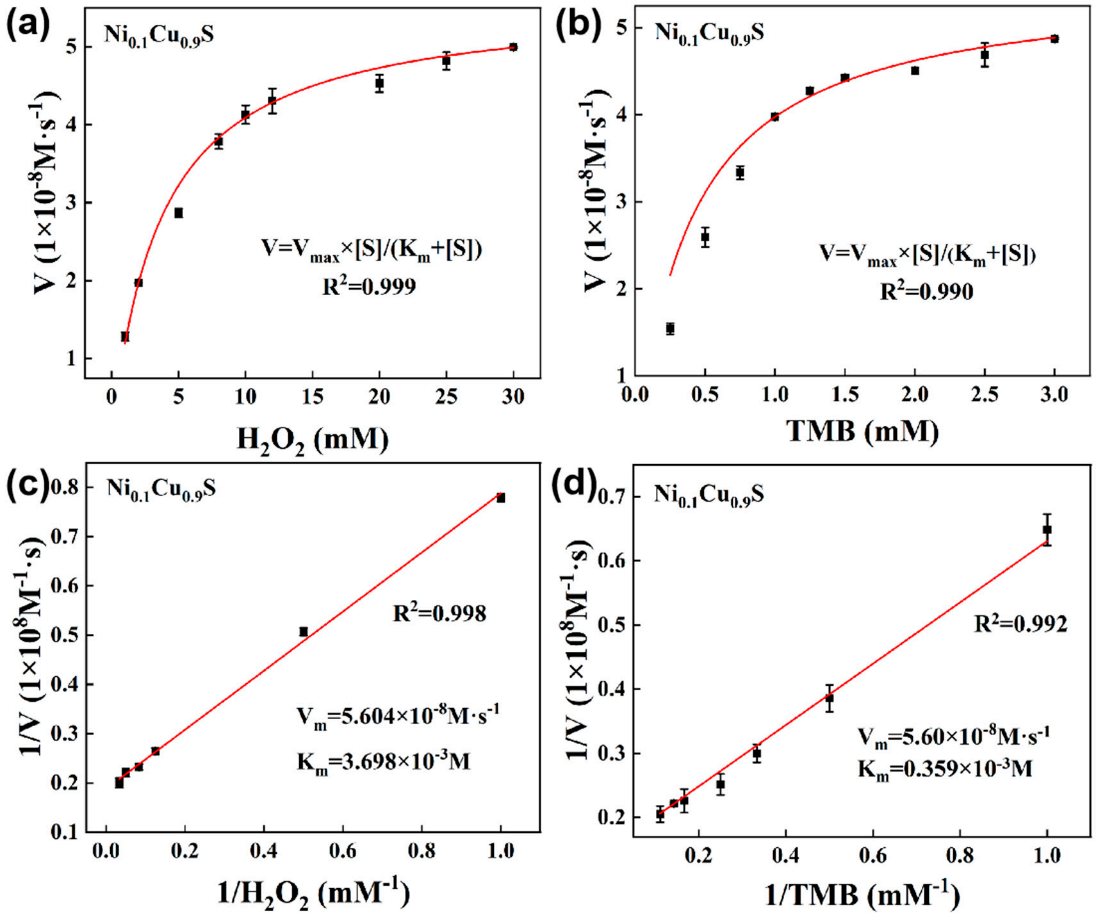

Figure S7. (a) and (b) the Michaelis-Menten curve for H_2O_2 and TMB, respectively. (c) and (d) the Line weaver-Burk plot for determination of kinetic constant of $Ni_{0.1}Cu_{0.9}S$ nanoflower for H_2O_2 and TMB, respectively.

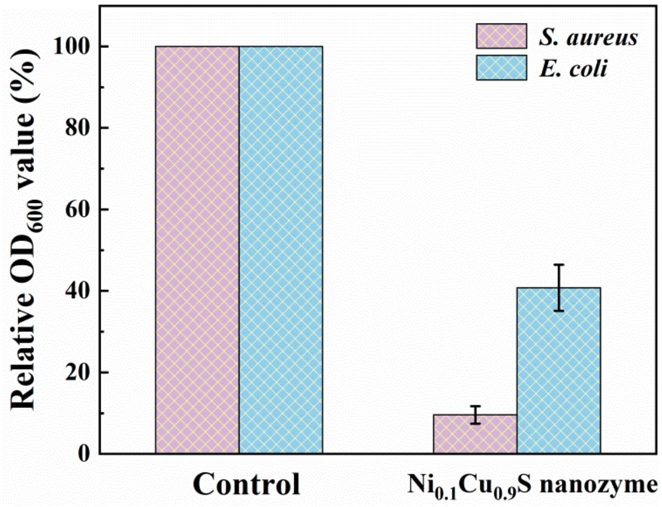

Figure S8. Relative OD_{600} values of $Ni_{0.1}Cu_{0.9}S$ nanozyme towards *E. coli* and *S. aureus*.

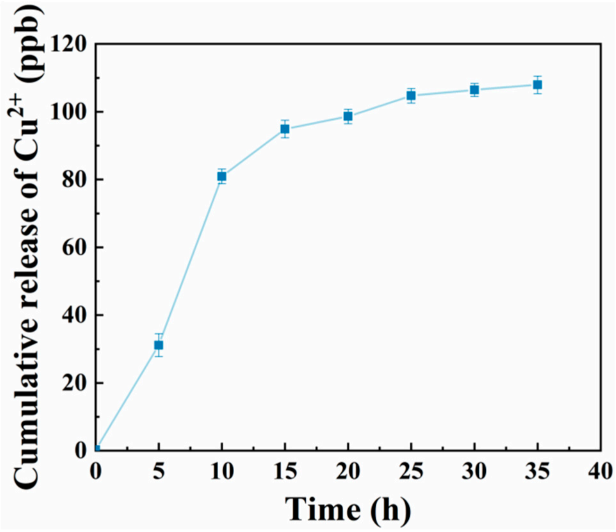

Figure S9. The release curve of time-dependent Cu^{2+} of $Ni_{0.1}Cu_{0.9}S$ nanozyme in test system plotted with data obtained by ICP.

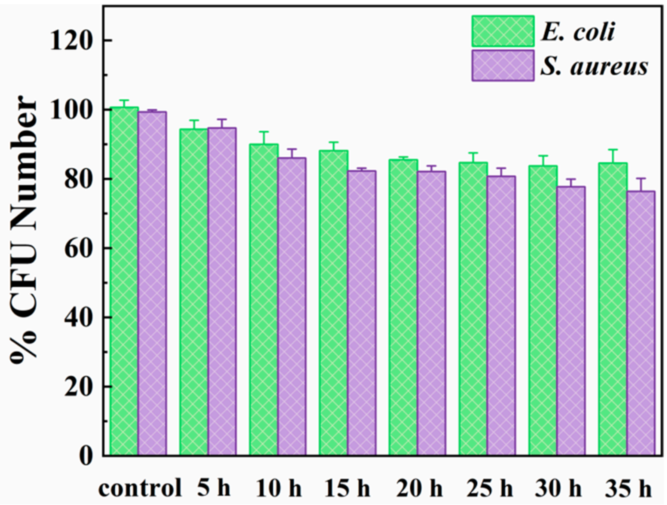

Figure S10. Survival rates of *E. coli* and *S. aureus* treated with Cu^{2+} supernatant samples.

Table S1. Summary of ICP-OES results for $Ni_{0.1}Cu_{0.9}S$ nanoflower.

Element	Wt.%	Atom ratio
Ni	5.97	Ni:Cu= 0.10:0.86
Cu	54.67	
S	8.25	

Table S2. Comparison of the K_m and V_{max} values for $Ni_{0.1}Cu_{0.9}S$ nanoflower with those of other peroxidase mimicase.

**Systems**	**Signal output**	**K_m/mM**		**V_{max}/(10-8 $M \cdot s^{-1}$)**		**Ref.**
		**TMB**	**H_2O_2**	**TMB**	**H_2O_2**	
TPyP-CuS	Colorimetry	0.106	3.937	5.30	2.139	[54]
CuS-BSA	Colorimetry	0.2	14	3.3	2	[55]
MXene/CuS	Colorimetry	0.072	2.08	4.63	6.34	[56]
CuS	Colorimetry	0.534	3.219	26.65	2.90	[57]
BNNS@CuS	Colorimetry	0.175	25	3.76	12.5	[58]
CuS-BSA-$Cu_3(PO_4)_2$	Colorimetry	0.33	0.29	16	8.31	[59]
PAN-CuO	Colorimetry	0.58	0.09	20.14	4.68	[27]
Fe_3O_4-SL	Colorimetry	0.374	0.208	6.298	4.561	[60]
Co_3S_4 nanosheets	Colorimetry	0.15	58.3	33	33	[61]
$NiCo_2S_4$(CTAB)NPs	Colorimetry	0.036	6.46	3.03	9.28	[62]
$NiCo_2S_4$(PVP)NPs	Colorimetry	0.175	7.43	7.97	19.0	[63]
Pt/$CuCo_2O_4$	Colorimetry	0.24	0.41	56.4	28.2	[63]
$Ni_{0.1}Cu_{0.9}S$	Colorimetry	2.541	7.293	10.98	5.409	This work

Table S3. The ICP-OES results of cumulative Cu^{2+} release from $Ni_{0.1}Cu_{0.9}S$ nanozyme in test system.

Time	0 h	5 h	10 h	15 h	20 h	25 h	30 h	35 h
Cu^{2+} Wt.%	0.00	0.24	0.63	0.74	0.77	0.81	0.83	0.84

Table S4. Comparison of different sensors for AA detection.

System	Signal output	Time	Stability	Linear range (μM)	LOD (μM)	Ref.
TPyP-CuS	Colorimetry	1min	No report	1-30	0.419	[54]
Pal@Co_3O_4 NCs	Colorimetry	-	No report	1-60	0.70	[59]
CD-QD@SiO2	Fluorescence	30min	No report	0-70	3.17	[64]
S, N-CDs/PVA	Fluorescence	2h	No report	10–500	6.99	[60]
Fe_3O_4@SiO_2-Ag NPs	Colorimetry	2min	No report	30-100	10.47	[65]
[Ni(HL)(bpe)1.5(H_2O)]	Fluorescence	30min	No report	2-10	1.23	[66]
FeMnzyme	Colorimetry	5min	No report	8-56	0.88	[67]
GA-AgNPs	Colorimetry	30min	No report	30-200	3.0	[68]
Pd-Pt-Ir	Colorimetry	3min	No report	25-800	11.7	[69]
$MnFe_2O_4$/MoS_2/SPCE	Electrochemical	-	No report	200-1000	175	[70]
AuNPs	Fluorescence	3min	No report	0-3000	130	[71]
$Ni_{0.1}Cu_{0.9}S$	Colorimetry	10min	30 days	10-800	0.84	This work

Table S5. Determination of the amounts of AA in real samples (n=3)

Sample	Added(uM)	Found(uM)	Recovery(%)	RSD(%,n=3)
Orange Juice	0	22.05	-	3.92
1	100	96.87	96.87	3.42
2	200	199.24	99.62	3.15
3	300	297.42	99.14	0.81
4	400	420.44	105.11	1.71
5	500	494.95	98.99	0.62

Notes and references

54. He, Y., Li, N., Lian, J., Yang, Z., Liu, Z., Liu, Q., Zhang, X., and Zhang, X. Colorimetric Ascorbic Acid Sensing from A Synergetic Catalytic Strategy Based on 5,10,15,20-Tetra (4-Pyridyl)-21H,23H-Porphyrin Functionalized CuS Nanohexahedrons with the Enhanced Peroxidase-like Activity, *Colloids Surf., A* **2020**, *598*, 124855.
55. Swaidan, A., Borthakur, P., Boruah, P. K., Das, M. R., Barras, A., Hamieh, S., Toufaily, J., Hamieh, T., Szunerits, S., and Boukherroub, R. A Facile Preparation of CuS-BSA Nanocomposite as Enzyme Mimics: Application for Selective and Sensitive Sensing of Cr(VI) Ions, *Sens. Actuators, B* **2019**, *294*, 253-262.
56. Li, Y., Kang, Z., Kong, L., Shi, H., Zhang, Y., Cui, M., and Yang, D.-P. MXene-Ti_3C_2/CuS Nanocomposites: Enhanced Peroxidase-like Activity and Sensitive Colorimetric Cholesterol Detection, *Mater. Sci. Eng. C* **2019**, *104*, 110000.
57. Tu, X., Ge, L., Deng, L., and Zhang, L. Morphology Adjustment and Optimization of CuS as Enzyme Mimics for the High Efficient Colorimetric Determination of Cr(VI) in Water, *Nanomaterials* **2022**, *12, 13423*.
58. Zhang, Y., Wang, Y.-N., Sun, X.-T., Chen, L., and Xu, Z.-R. Boron Nitride Nanosheet/CuS Nanocomposites as Mimetic Peroxidase for Sensitive Colorimetric Detection of Cholesterol, *Sens. Actuators, B* **2017**, *246*, 118-126.
59. Swaidan, A., Barras, A., Addad, A., Tahon, J.-F., Toufaily, J., Hamieh, T., Szunerits, S., and Boukherroub, R. Colorimetric Sensing of Dopamine in Beef Meat using Copper Sulfide Encapsulated within Bovine Serum Albumin Functionalized with Copper Phosphate (CuS-BSA-$Cu_3(PO_4)_2$) Nanoparticles, *J. Colloid Interface Sci.* **2021**, *582*, 732-740.
27. Zheng, X., Lian, Q., Zhou, L., Jiang, Y., and Gao, J. Peroxidase Mimicking of Binary Polyacrylonitrile-CuO Nanoflowers and the Application in Colorimetric Detection of H_2O_2 and Ascorbic Acid, *ACS Sustainable Chem. Eng.* **2021**, *9*, 7030-7043.
60. Liu, H.-Y., Xu, H.-X., Zhu, L.-L., Wen, J.-J., Qiu, Y.-B., Gu, C.-C., and Li, L.-H. Colorimetric Detection of Hydrogen Peroxide and Glutathione Based on Peroxidase Mimetic Activity of Fe_3O_4-sodium Lignosulfonate Nanoparticles, *Chinese J. Anal. Chem.* **2021**, *49*, e21160-e21169.
61. Hashmi, S., Singh, M., Weerathunge, P., Mayes, E. L. H., Mariathomas, P. D., N. Prasad, S., Ramanathan, R., and Bansal, V. Cobalt Sulfide Nanosheets as Peroxidase Mimics for Colorimetric Detection of l-Cysteine, *ACS Appl. Nano Mater.* **2021**, *4*, 13352-13362.
62. Lian, M., Liu, M., Zhang, X., Zhang, W., Zhao, J., Zhou, X., and Chen, D. Template-Regulated Bimetallic Sulfide Nanozymes with High Specificity and Activity for Visual Colorimetric Detection of Cellular H_2O_2, *ACS Appl. Mater. Interfaces* **2021**, *13*, 53599-53609.
63. Xue, Y., Li, H., Wu, T., Zhao, H., Gao, Y., Zhu, X., and Liu, Q. Pt Deposited on Sea Urchin-like $CuCo_2O_4$ Nanowires: Preparation, the Excellent Peroxidase-like Activity and the Colorimetric Detection of Sulfide Ions, *J. Environ. Chem. Eng.* **2022**, *10*, 107228.
64. Zhao, T., Zhu, C., Xu, S., Wu, X., Zhang, X., Zheng, Y., Wu, M., Tong, Z., Fang, W., and Zhang, K. Fluorescent Color Analysis of Ascorbic Acid by Ratiometric Fluorescent Paper Utilizing Hybrid Carbon Dots-Silica Coated Quantum dots, *Dyes Pigm.* **2021**, *186*, 108995.
65. Tarighat, M. A., Ghorghosheh, F. H., and Abdi, G. Fe_3O_4@SiO_2-Ag Nanocomposite Colorimetric Sensor for Determination of Arginine and Ascorbic Acid Based on Synthesized Small Size AgNPs by Cystoseria Algae Extract, *MATER SCI ENG B-ADV* **2022**, *283*, 115855.
66. Wang, Y.-N., Wang, S.-D., Fan, Y., Yu, L., Zha, R.-H., Liu, L.-J., Wen, L.-M., Chang, X.-P., Liu, H.-Q., and Zou, G.-D. A Dual-Chemosensor Based on Ni-CP: Fluorescence Turn-on Sensing toward Ascorbic Acid and Turn-Off Sensing toward Acetylacetone, *J. Lumin.* **2022**, *243*, 118680.
67. Han, Y., Luo, L., Zhang, L., Kang, Y., Sun, H., Dan, J., Sun, J., Zhang, W., Yue, T., and Wang, J. Oxidase-like Fe–Mn Bimetallic Nanozymes for Colorimetric Detection of Ascorbic Acid in Kiwi Fruit, *LWT* **2022**, *154*, 112821.
68. Doan, V.-D., Nguyen, V.-C., Nguyen, T.-L.-H., Nguyen, A.-T., and Nguyen, T.-D. Highly Sensitive and Low-Cost Colourimetric Detection of Glucose and Ascorbic Acid Based on Silver Nanozyme Biosynthesized by Gleditsia Australis Fruit, *Spectrochim. Acta, Part A* **2022**, *268*, 120709.
69. He, J., He, D., Yang, L., Wu, G.-L., Tian, J., Liu, Y., and Wang, W. Preparation of Urchin-like Pd-Pt-Ir Nanozymes and Their Application for the Detection of Ascorbic Acid and Hydrogen Peroxide, *Mater. Lett.* **2022**, *314*, 131851.
70. Wu, P., Huang, Y., Zhao, X., Lin, D., Xie, L., Li, Z., Zhu, Z., Zhao, H., and Lan, M. $MnFe_2O_4$/MoS_2 Nanocomposite as Oxidase-like for Electrochemical Simultaneous Detection of Ascorbic Acid, Dopamine and Uric Acid, *Microchem. J.* **2022**, *181*, 107780.
71. Lin, S., Liu, S., Dai, G., Zhang, X., Xia, F., and Dai, Y. A Click-Induced Fluorescence-Quenching

Sensor Based on Gold Nanoparticles for Detection of Copper(Ⅱ) Ion and Ascorbic Acid, *Dyes Pig.* **2021**, *195*, 109726.
